# Effectiveness of Mindfulness-Based Cognitive Therapy (MBCT) in Reducing Aggression of Individuals at the Juvenile Correction and Rehabilitation Center

**DOI:** 10.5812/ijhrba.14818

**Published:** 2013-12-24

**Authors:** Atefeh Milani, Zahra Nikmanesh, Ali Farnam

**Affiliations:** 1 Department of Psychology, Faculty of Education and Psychology, University of Sistan and Baluchestan, Zahedan, IR Iran

**Keywords:** Mindfulness, Aggression, Juvenile, Rehabilitation Centers

## Abstract

**Background:**

In the present era, delinquency in children and adolescents is undoubtedly a difficult and upsetting issue attracting the attention of many experts such as psychologists, sociologists, and criminologists. These experts often try to answer why a number of children and adolescents engage in various crimes such as aggressive and anti-social crimes. They also try to find out how these crimes can be prevented.

**Objectives:**

The present study investigates the effectiveness of mindfulness-based cognitive therapy training (MBCT) in reducing aggression in a juvenile correction and rehabilitation center of Zahedan province during years 1991 to 1992.

**Materials and Methods:**

This experimental study included an experimental and a control group with a pretest, posttest, and follow-up approach. The Buss and Perry aggression questionnaire (1992) was used for data collection. The sample group included 22 (10 experimental and 12 control groups) adolescent males in a juvenile correction and rehabilitation center of Zahedan province who were selected through a census method. Using a matching method based on the pre-test scores of the aggression questionnaire, they were then divided into two equivalent categories and were randomly assigned to the two groups. Mindfulness-based cognitive training took the group training in 8 sessions administered on experimental group. The follow-up test was conducted two weeks after the end of the posttest sessions. The results were analyzed using ANCOVA.

**Results:**

The results of ANCOVA showed that mindfulness-based cognitive training could significantly reduce aggression during posttest and follow-up test phases in the experimental group, compared to the control group (P < 0.01). Moreover, the results indicated the effectiveness of this method in significantly reducing anger, physical aggression, and hostility during posttest and follow-up test phases (P < 0.05). However, no significant reduction was observed in the verbal aggression subscale.

**Conclusions:**

According to the results of the present study, mindfulness-based cognitive training seems to be effective for reducing aggressive behaviors.

## 1. Background

Acts of violence and aggressive behavior have long been common in human societies. Referring to figures in articles and scientific resources warns us of violence and conflicts that result in a high rate of murder and suicide (as the most severe forms of external and internal aggressions, respectively). There have been two trends in aggressive behavior during the recent years. The first one is increased aggression in different social groups and decrease in the average age of aggressive individuals so that adolescents were involved in most beatings. The second one is an increase in such behaviors at schools. Uncontrolled aggression causes social, occupational, educational, physical and mental health problems among adolescents. It is also a predictor of alcohol and drug use, smoking, low adaptability at school, educational failure, depression, delinquency, and other disorders among adolescents (1).

Given the extent, incidence, and unpleasant effects of aggressive behavior, anger and aggression have long been considered as a problem needing clinical and legal investigations. Some treatment methods for aggression include cognitive therapy, cognitive-behavioral therapy, hybrid programs, gestalt therapy, abreaction, development of social skills, and medical therapy. As a new therapeutic method, mindfulness therapy may reduce aggression as it trains cognitive skills for the management of aggressive behavior (2).

Originating from Eastern meditation, mindfulness is described as a method to pay attention (full attention) to the experience of the present moment. Mindfulness based cognitive therapy (MBCT) was devised by Segal, Williams, and Teasdale (3) as an 8-week program with group-held sessions mainly based on the mindfulness-based stress reduction (MBSR ) program devised by Kabbat-Zinn (4). This program encompasses some elements of the cognitive therapy that separate an individual’s view from his/her thoughts (e.g. statements such as “thoughts are not facts” and “I am not my thoughts”). This type of cognitive therapy includes different meditations, stretching yoga, introduction to depression, workout review, and a few cognitive therapy exercises that show the relationship between mood, thoughts, behavior, and feelings as well as emotions and physical sensations (5).

Dialectical behavior therapy, acceptance and commitment therapy, substance abuse relapse prevention, MBSR, and MBCT are among interventions encompassing mindfulness training (6). Similarly, mindfulness based cognitive therapy might be used for cognitive behavioral therapy, which is considered as a valid therapy for the management of anger and aggression. There is some evidence indicating that mindfulness reduces physical and mental problems. These problems include chronic pain and depression relapse (7), mood and stress disorder (8), and anxiety. Until present, however, several attempts investigating the effectiveness of this approach on anger and aggression have failed (9).

The observation without judgment provides the opportunity to recognize the consequences of a certain behavior (e.g. irritating the boss with frequent delays). This recognition will lead to more effective behavioral changes. Nonetheless, the aim of mindfulness training is to learn to observe without making a judgment about the current conditions; the conditions that may result in automatic nervous system arousal, competitive thoughts, muscle tension, and other phenomena incompatible with relaxation (8). Taking advantage of mindfulness-based cognitive therapy and other methods associated with it, many empirical studies have been carried out for the treatment of many diseases and mental disorders.

Studies on the effectiveness of mindfulness-based cognitive therapy in reducing aggression and anger among drivers (10) and married males (11) showed a significant decrease in anger and aggression and increased self-control in aggressive behavior (12). All the participants reported that mindfulness-based cognitive therapy had a positive effect on their life and the way they coped with their anger (9). Moreover, effectiveness of mindfulness-based cognitive therapy in reducing depression and anxiety (13) and among suicidal depressed patients (14) showed that MBCT reduces negative automatic thoughts and dysfunctional attitudes, increases life interest, desire to survive, coping with life’s problems, and improves family, educational, and occupational functioning among suicidal depressed patients.

Aggression is considered as a major problem in human relationships for the following reasons:

1. uncontrolled aggression causes social, occupational, educational, and physical and mental health problems for adolescents.
2. It is also a predictor of alcohol and drug use, smoking, low adaptability at school, educational failure, depression, delinquency, and other disorders (15).
3. It increases the rate of violent crime among adolescents, domestic abuse, racial differences, and recent acts of terrorism (16).

A group of specialists argued that depression, physical complaints and aggression, in juvenile delinquents are two times more than that of other youths. Moreover, not only the delinquents with mental disorders committed more faults in the jail, but also they were more likely to be victimized by the others (17).

Given the prevalence of aggressive behavior and defects of various therapeutic methods, and since many cohort studies (18, 19) were reported to have weak designs and limited credibility (2); and with regards to the success of mindfulness-based cognitive therapies; and on the other hand results of research on jail and crime preventative efforts showing that not only penalty and punishment cannot prevent aggression and crime, but also they sometimes increase their intensity and number (17); thus the present study was conducted to partially address these issues.

## 2. Objectives

The aim of this study was to determine the effectiveness of mindfulness-based cognitive therapy in reducing aggression and to find whether mindfulness-based cognitive training reduces aggression among adolescents.

## 3. Materials and Methods

This experimental study included two experimental and control groups with a pretest, posttest, and follow-up. The statistical population included 38 adolescent males in juvenile correction and rehabilitation center of Zahedan province during 1991-1992 years. The sample comprised of 22 subjects whom were selected through the census method. They were assigned into two groups, 10 subjects in the experimental and 12 subjects in the control group based on the scores of the pretest. All participants were interested in participating in the research project and workshop. The experimental group was trained. The following instruments were used for the data collection.

### 3.1. Questionnaire

This questionnaire test was devised by Buss and Perry (20). The questionnaire included 29 items and 4 subscales measuring aggression. The options were scaled from 1 (it does not describe me perfectly) to 5 (it describes me perfectly). The results show that the questionnaire has good internal consistency. Moreover, the correlation of the questionnaire subscales between each other and the total scale which varies between 0.25 and 0.45. This questionnaire's reliability was reported by Fives, Kong, Fuller and Digiuseppe (2011); 0.94 for sum of subscales of aggression, 0.88 for physical aggression, 0.76 for verbal aggression, 0.78 for anger, and 0.82 for hostility (21). Mohammadi (2006) also examined this questionnaire by three different methods; the reliability was calculated as 0.89, 0.78, and 0.73 by the Coranbach alpha, Test-retest, and Split-half methods respectively indicating the appropriate validity of this instrument (22). In the present study, the alpha coefficient for the total score of the questionnaire was 0.73.

### 3.2. Methods

For the experimental group, eight 1.5-hour mindfulness training sessions were held at the center, two days a week. Instructions for each session were organized based on the book of “mindfulness based cognitive therapy” (13). The control group received no training. At the end of the last session, the subjects completed the questionnaire. After two weeks, the subjects of both groups, once again, answered the questionnaire at the center. The content of the sessions was as follows: first session: distribution of the questionnaire, introduction, outlines presentation and objectives of the sessions, clients’ familiarity with the logic of attention control training, explaining the relationship of ABC. Second session: three-minute breathing, body checking practice. Third session: breathing and mindfulness of breathing exercise. Fourth session: wandering mind and invasion of thoughts, concentration, and clouds technique. Fifth session: seated meditation practice, explaining how to control the mind, controlling negative thoughts, accepting thoughts and feelings without judgment. Sixth session: positive thoughts and mind control practice, mindfulness of sounds and train station technique. Seventh session: mindfulness walking practice. Eighth session: summarizing what was learned, distribution of the questionnaire, ending the class with the last meditation. A similar training workshop was held for the control group after the research was complete.

## 4. Results

The data obtained were analyzed using the SPSS software. The difference between the two research groups was studied using inferential statistics (analysis of covariance and independent t-test). Table 1 shows the mean and standard deviation of the total scores of aggression and its subscales for the pre-test, post-test, and follow-up phases.

**Table 1.** Mean and Standard Deviation of the Total Scores of Aggression and Its Subscales for the Pretest, Posttest, and Follow-up Phases in Two Groups

Variables	Pre-test, Mean ± SD	Post-test, Mean ± SD	Follow-Up, Mean ± SD
**Aggression**			
Experimental	85.40 ± 18.48	69 ± 13.24	75.1 ± 8.38
Control	88.33 ± 11.38	89.50 ± 18.20	92.75 ± 16.90
**Anger**			
Experimental	20.70 ± 3.56	16.70 ± 3.62	18.30 ± 2.49
Control	20.33 ± 3.68	22 ± 4.45	22.25 ± 4.43
**Verbal aggression**			
Experimental	14.80 ± 3.22	12.4 ± 2.59	13.30 ± 2
Control	15.33 ± 2.90	13.58 ± 3.72	14.25 ± 3.51
**Physical aggression**			
Experimental	26.80 ± 7.56	21.20 ± 4.96	23.50 ± 3.02
Control	29.33 ± 5.08	27.83 ± 6.27	29.50 ± 6.02
**Hostility**			
Experimental	23.10 ± 7.18	18.70 ± 3.52	20 ± 2.26
Control	22.83 ± 5.96	26.08 ± 7.37	26.75 ± 7.02

As the Table 1 shows, the aggression score of the control group had the highest mean (m = 88.33) in the pretest phase. Moreover, in the control group, the highest mean aggression score in the posttest and follow-up phases was 89.50 and 75.92, respectively. The verbal aggression subscale of the experimental group had the lowest mean (80.14) in the pretest phase. After that, the verbal aggression subscale of the experimental group had the lowest mean in the posttest and follow-up phases (m = 40.12 and m = 30.13), respectively.

The t-test for independent groups was used to examine the difference between the experimental and control groups in terms of their aggression score and its subscales in the pretest phase. Table 2 shows the results.

**Table 2.** Independent T-test Results; Comparing the Experimental and Control Groups in Terms of Their Total Scores of Aggression and Its Subscales

Pre-test	Mean ± SD	t	f	sig
**Aggression**		-0.45	2.64	0.65
Experimental	85.40 ± 18.48			
Control	88.33 ± 18.38			
**Anger**		-0.08	0.01	0.93
Experimental	20.70 ± 3.56			
Control	20.83 ± 3.68			
**Verbal aggression**		-0.93	2.42	0.36
Experimental	26.80 ± 7.56			
Control	29.33 ± 5.08			
**Physical aggression**		-0.40	0.74	0.68
Experimental	14.80 ± 3.22			
Control	15.33 ± 2.90			
**Hostility**		0.09	2.18	0.92
Experimental	23.10 ± 7.18			
Control	22.83 ± 5.96			

As indicated, no significant difference was observed between the mean scores of aggression and its subscales in the experimental and control groups in the pretest phase.

Levene’s test showed that there is no significant difference between the two groups in terms of their variances in aggression, anger and verbal aggression. Therefore, after controlling the pretest variable, analysis of covariance was used in order to compare the mean difference between the two groups in posttest and follow-up phases. Table 3 shows the results of the analysis of covariance.

**Table 3.** The Results of the Analysis of Covariance in Aggression, Anger and Verbal Aggression Scores

	Sum of Square	df	Coefficient F	(P)	Effect Size	Statistical Power
**Aggression in post-test**						
Pre-test	1.53	1	0.00	0.94	0.00	0.05
Group	2280.60	1	8.29	0.01	0.30	0.78
**Aggression in follow up**						
Pre-test	0.75	1	0.00	0.95	0.00	0.05
Group	1688.63	1	8.50	0.00	0.30	0.79
**Anger in posttest**						
Pre-test	0.14	1	0.00	0.93	0.00	0.05
Group	152.98	1	8.65	0.00	0.31	0.79
**Anger in follow up**						
Pre-test	0.11	1	0.00	0.93	0.00	0.05
Group	85.19	1	5.94	0.02	0.23	0.63
**Verbal aggression in posttest**						
Pre-test	14.22	1	1.35	0.25	0.06	0.19
Group	5.80	1	0.55	0.46	0.02	0.10
**Verbal aggression in follow up**						
Pre-test	6.80	1	0.78	0.38	0.03	0.13
Group	3.89	1	0.44	0.51	0.02	0.09

As Table 3 shows, there was a significant difference between the experimental and control groups in terms of mean aggression after controlling the pretest variable in posttest and follow-up phases (P < 0.01). There was a significant difference between the experimental and control groups in terms of mean anger after controlling the pretest variable in Post-test (P < 0.01) and follow-up phases (P < 0.05). There was no significant difference between the mean verbal aggression subscale in posttest and follow-up phases.

Due to the significance of Levene’s test in physical aggression and hostility subtest, it was not appropriate to use the analysis of covariance. Therefore, after obtaining the difference between pretest and posttest scores, an independent group t-test was used and the following results were obtained.

**Table 4.** Comparing the Change in Physical Aggression and Hostility in Posttest and Follow-up Phases^a^

	Mean ± SD	t	f	sig
**Physical aggression in post-test**		-2.70	1.11	0.01
Experimental	20.21 ± 4.96			
Control	83.27 ± 6.27			
**Physical aggression in follow up**		-3.02	5.63	0.00
Experimental	50.23 ± 3.02			
Control	50.29 ± 6.02			
**Hostility in post-test**		-3.07	4.26	0.00
Experimental	70.18 ± 3.52			
Control	08.26 ± 7.37			
**Hostility in follow up**		-3.14	4.78	0.00
Experimental	20 ± 2.26			
Control	75.26 ± 7.02			

^a^ Number of participants in experimental group = 10; Number of participants in control group = 12.

Table 4 shows, that there was a significant difference between the experimental and control groups in Post-test (P < 0.05) and follow-up phases (P < 0.01) in terms of the physical aggression subscale. Moreover, there was a significant difference between the two groups in Post-test and follow-up phases in terms of the hostility subscale (P < 0.01).

## 5. Discussion

The results of the present study showed that mindfulness-based cognitive training has been effective in reducing aggression. This finding is consistent with the results of previous (2, 10, 18, 19, 23-25) studies looking at the reduction of aggressive behavior after mindfulness-based interventions. These results were also consistent with the results of other research (9-11) on the effectiveness of mindfulness-based cognitive therapy in reducing aggression. These results were inconsistent with the results of Singh et al. (26), and consistent with the results of the present study on the effectiveness of training in reducing verbal aggression among people with mild cognitive impairment. Due to its stressful nature, loss of freedom, deep psychological trauma, long-term absence from family and society, the prison environment is by far different from other social and correctional environments. The reason why verbal aggression is not reduced might be attributed to prison environment and random factors beyond experimental conditions. Moreover, compared to other aspects of aggression such as physical aggression, verbal aggression is considered as a normal behavior among prisoners. The results of the present study were consistent with the results of Chilver et al. (18) and Murphy (23) on the effectiveness of MBSR in reducing hostility. These findings confirm the prevailing view that people who have mindfulness are less likely to respond to others’ aggression, threat, and insult aggressively (9, 27). Mindfulness-based therapies encourage clients to learn concentration, non-judgment and acceptance, and live in the present. Mindfulness-based therapy offers cognitive skills, which lead to a change in cognition. This therapy, is unique since it is not dependent on a second participant and is mostly preferred by clients with aggressive problems (2). Studies show that mindfulness-based therapy makes people to be more exposed to anger-provoking stimuli by reducing anger and maladaptive responses. It also facilitates cognitive changes and helps increase self-regulation ability through the process of encouraging clients to view emotions such as anger as a temporary emotion, not as the consequences of a certain behavior. Mindfulness trains people to accept the fact that anger is currently felt and it takes time to consider how to respond to it. If clients’ immediate response to anger is to act angrily, mindfulness can lead to a more adaptive response. Finally, mindfulness has much to offer for the treatment of aggression and anger, and it is capable of promoting comfort and flexibility through the development of positive emotional states. One of the limitations of the present study is the lack of similar studies on mindfulness and aggression, which makes results comparison impossible. Subject attrition and short duration of the follow-up test (because the subjects were released) are among the other limitations of this study. It is recommended that similar studies on other groups outside the prison environment including students, women, girls, and children should be performed with longer follow-up test for at least 6 months.
